# Epidemiology of laboratory confirmed measles virus cases in Amhara Regional State of Ethiopia, 2004–2014

**DOI:** 10.1186/s12879-016-1457-7

**Published:** 2016-03-22

**Authors:** Mekonen Getahun, Berhane Beyene, Ayesheshem Ademe, Birke Teshome, Mesfin Tefera, Anjelo Asha, Aklog Afework, Yoseph HaileMariyam, Esete Assefa, Kathleen Gallagher

**Affiliations:** Ethiopian Public Health Institute, Arbegnoch Street, P. O. Box 1242, Addis Ababa, Ethiopia; WHO Ethiopia Country Office, Addis Ababa, Ethiopia

**Keywords:** Laboratory Confirmed Measles, Amhara Regional State of Ethiopia

## Abstract

**Background:**

Measles is a highly contagious viral infection causing large outbreaks all over the world. Despite the availability of safe and cost effective vaccine, measles remained endemic with persistent periodic outbreaks in the Horn of Africa. The aim of this study is to characterize laboratory confirmed measles cases in Amhara Regional State, which was one of the highly affected regions in Ethiopia.

**Method:**

A suspected measles case was defined as any person presenting with fever, maculopapular rash and one or more of the three symptoms cough, coryza or conjunctivitis or a patient in whom a clinician suspects measles. A blood sample was collected for any measles suspected patient with a case based investigation form and specimen transported to the National Measles Laboratory in good condition where it was to be tested for Measles IgM antibody by ELISA technique. Data was entered and analyzed using Epi-Info 3.5.4 software.

**Result:**

A total of 6579 samples were tested for measles IgM among 7296 samples collected in Amhara Regional State over 11 years (2004–2014). Of the tested samples, 2412 (36.7 %) were found positive, while 3965 and 202 samples were found to be negative and equivocal (compatible) respectively. Patients with age ≥10 years were the most affected. The highest number of laboratory confirmed measles cases were detected in 2014 and cases were occurred in all of the 11 zones of the state. A seasonal peak was noted in the hot-dry season of the year.

**Conclusion:**

Measles remains to be a public health problem in Amhara Regional State of Ethiopia, mostly affecting people ≥10 years of age. Measles virus was detected in all zones of the state, reaching its peak in the hot-dry season. To reduce the incidence of measles, it is highly recommended to improve routine immunization, and conduct a wide age group campaign.

Additional research to evaluate the knowledge, attitudes and practices of the general population and health care professionals about measles infection and vaccination is important. Genotyping of circulating measles virus strain is recommended.

## Background

Measles is one of the most contagious vaccine preventable viral diseases and is known to cause severe complications such as encephalitis, pneumonia, ear and sinus infections, persistent diarrhea, upper airway obstruction, mouth ulcers and death. It is caused by the genus Morbillivirus within the family Paramyxovirus for which humans are the only reservoir [1]. Transmission is primarily person-to-person via aerosolized droplets or by direct contact with the nasal and throat secretions of infected persons. Measles outbreaks occur when the number of susceptible individuals in the population reaches a critical threshold due to low vaccination coverage and high prevalence of malnutrition [2].

Globally, an estimated 197,000 measles deaths occurred in 2007, with 136,000 (69 %) and 45,000 (23 %) occurring in Southeast Asia and Africa respectively. Generally in Africa, the measles case fatality rate ranges from 3 to 5 %, reaching up to 30 % during severe outbreaks and outbreaks in closed communities such as refugee camps [3]. Measles continues to be a major public health problem in Africa, causing an estimated 28,000 deaths each year [4].

Following the initiation of accelerated measles control, Ethiopia has conducted a series of emergency and catch-up measles immunization campaigns between 2002 and 2004 which were conducted in a phased manner throughout the country, with average national coverage of 92 %. Measles case based surveillance, including laboratory confirmation, was initiated in 2004 to enhance detection, investigation and control of outbreaks. Since 2005 sub-national level follow-up SIAs have been carried out in an interval of 2–4 years [5, 6].

Among others the case-based surveillance for measles requires that all suspected measles cases are investigated during initial contact with the case using an individual case investigation form; a blood specimen is collected for serologic confirmation of measles infection. The laboratory plays a central role in the confirmation of suspected cases and outbreaks, and in the identification of circulating strains of measles virus [2].

The WHO African measles elimination goals aim at improving case based surveillance and achieving and sustaining high measles vaccination coverage to reduce measles incidence < 1 confirmed cases per million by 2020 in the African region including Ethiopia [2]. But in recent years, persistent and recurrent measles outbreaks have occurred in several areas of Ethiopia including Amhara Regional State and more than 14,000 confirmed measles cases were reported in Ethiopia during 2014 [7–9]. The purpose of this report is to characterize the epidemiology of laboratory-confirmed measles cases in the Amhara Regional State of Ethiopia and to provide evidence for the decision making process in an effort to eliminate measles in Ethiopia.

## Methods

### Study setting

**Fig. 1:**
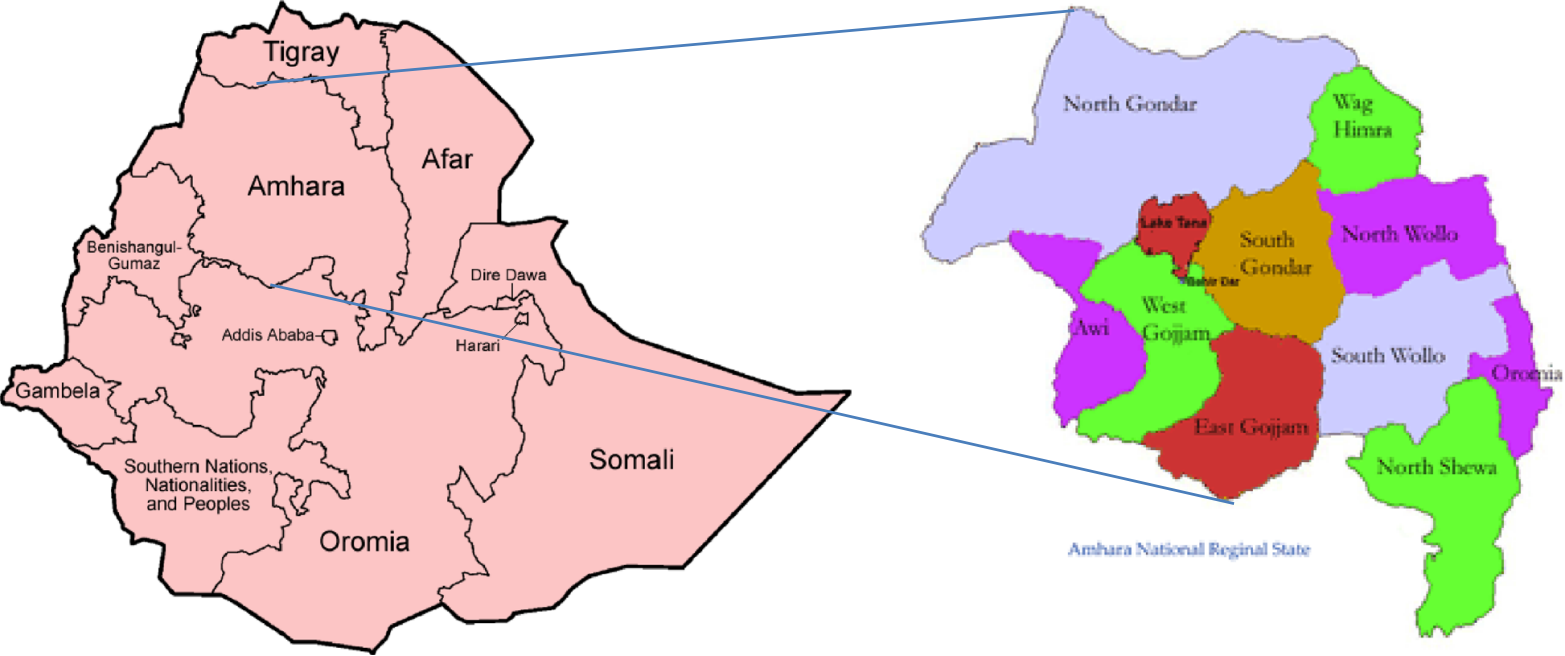
Map of Ethiopia Showing the Relative Location of Amhara Regional State and its Administrative Zones (Source, WIKIPEDIA, the Free Encyclopedia)

The study was conducted in the Amhara Regional State, the second most populous region in Ethiopia with a population of 17.22 million [10]. The region covers an area of 154,709 km^2^ and is located in the North Western and North central part of Ethiopia. The State shares common borders with Tigray, Afar, Oromia and Benshangul Gumuz regional states in North, East, South and South-West respectively and the Republic of South Sudan in the west. Administratively the region is divided into 11 zones and further sub-divided into woredas and kebeles (Fig. 1).

### Study population and case definitions

National guidelines for measles surveillance are based on the WHO/-AFRO Measles and Rubella Surveillance guideline, and define a suspected measles case as any person with a generalized maculopapular rash and fever plus one or more of the following: 1) cough, 2) coryza), or 3) conjunctivitis OR any person in whom a clinician suspects measles. A case of laboratory confirmed measles is a suspected measles case that is investigated (including the collection of blood specimen), and has serological confirmation of recent measles virus infection (presence of measles IgM antibody) who has not received measles vaccination in the 30 days preceding the specimen collection. Measles confirmed by epidemiological linkage is a suspected measles case that has not had a specimen taken for serologic confirmation and is linked (in place, person and time) to a lab confirmed cases. A clinically confirmed/compatible measles case is defined as a suspected measles case that has not had a blood specimen taken for serologic confirmation and is not linked epidemiologically to any lab confirmed outbreak of measles. A confirmed outbreak of measles is defined as 3 or more laboratory confirmed cases of measles in a health facility or district in a one month period [2].

All patients regardless of age and sex that fulfilled the case definition for measles during the study period (2004–2014) were included in this analysis.

### Specimen collection and transportation

Between 1 January 2004 and 31 December 2014, blood samples were collected from suspect measles cases in all zones of Amhara Regional State and transported to the National Measles Laboratory, located at the Ethiopian Public Health Institute (EPHI) in Addis Ababa for laboratory confirmation. Samples were collected and transported in good laboratory condition (adequate volume, no leakage, not turbid from possible contamination, not desiccated and container temperature of 2–8°c). Demographic and clinical information about the patient was captured through the case based investigation form.

### Laboratory method and quality assurance

Samples were tested for measles virus specific IgM antibody by indirect enzyme linked immuno-sorbent assay (ELISA) using a commercially available test kit (Siemens Healthcare Diagnostics Products GmbH, Marburg, Germany). Standard operational procedures (SOPs), and job aids are available for the laboratory activities. All the instruments and materials of the laboratory were supplied by WHO.

The Ethiopian National Measles Laboratory is a member of the global WHO vaccine-preventable disease laboratory network and works according to WHO standards and protocols, receives external quality assessment (EQA) panel samples annually, sends 10 % quality control (QC) samples to regional reference laboratory (RRL) quarterly and is accredited annually. The laboratory also applied external (kit) and internal (in house) control samples in each run and results were reported only when the run was valid based on the controls. The national laboratory scored ≥95 % performance for both EQA and QC during this study period.

### Data analysis and ethical consideration

The laboratory test result and demographic information provided on the case investigation form were entered into the measles case-based surveillance system database. Data were consolidated, cleaned, analyzed and disseminated to stake holders for action using Epi-Info software version 3.5.4. Demographic characteristics of reported cases and trends in cases reported over time were assessed. The study was ethically approved by institutional review board of EPHI.

## Results

Between 01 January 2004 and 31st December 2014, 7296 suspected measles cases investigated had both case report forms and blood samples available through the case-based surveillance system. Of these cases, 3781 (51.2 %) were males. The information on gender was unavailable for 17 cases. The mean age of suspected cases was 9.7 years, ranging from a month to 68 years (SD = ±8.8 years).

**Fig. 2:**
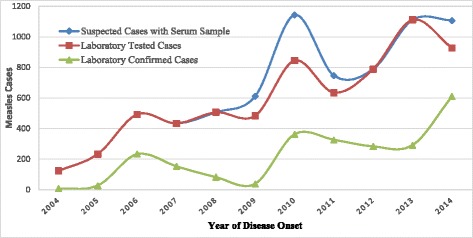
Trend of Measles Suspected, Laboratory Tested and Confirmed Cases by Year of Disease Onset in Amhara Regional State of Ethiopia, 2004–2014

**Fig. 3:**
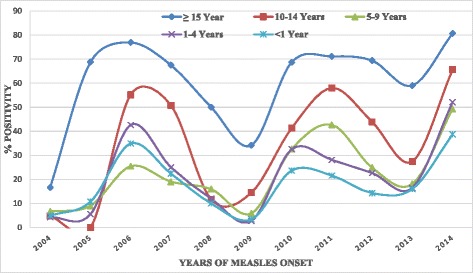
Distribution of Laboratory Confirmed Measles cases by Year and Month of disease onset in Amhara Regional State of Ethiopia, 2004–2014

Measles was detected in all 11 zonal administrations with the lowest number of cases (75) and positivity rate (3.1 %) in Bahir Dar special zone. Higher number of cases and positivity rate were noted in North Gonder (394, 16.3 %) and South Wello (392, 16.3 %) zones (Table 1). The suspected cases increased from 123 in 2004 to 1107 in 2014 (Fig. 2). Measles cases were reported throughout the year with peaks in February (294) and March (348) and the lower number (100) of cases reported in August (Fig. 3).

**Table 1:** Distribution of confirmed measles cases by selected demographic characteristics of patients, Amhara Regional State, Ethiopia, 2004–2014

Characteristics	Number of confirmed Measles cases	% of confirmed Measles cases
Sex	*N* = 2412	
Male	1251	51.9
Female	1152	47.8
Missed	9	0.3
Zones	*N* = 2412	
Awi	122	5.1 %
Bahir Dar	75	3.1 %
East Gojjam	240	10.0 %
North Gonder	394	16.3 %
North Shewa	381	15.8 %
North Wello	164	6.8 %
Oromiya	103	4.3 %
South Gonder	241	10.0 %
South Wello	392	16.3 %
West Gojjam	221	9.2 %
Wag Himra	79	3.3 %
Age Group (Years)	*N* = 2412	
<1	91	3.8 %
1–4	429	17.8 %
5–9	348	14.4 %
10–14	366	15.2 %
= > 15	1160	48.1 %
Missing	18	0.7 %

Of the 7296 suspected cases, 6579 (90 %) samples were tested by the laboratory for measles virus specific IgM antibody. Of these 2412 (36.7 %) were IgM positive (laboratory confirmed measles): 3965 cases were negative for the presence of measles IgM antibody and 202 specimens had equivocal lab results.

**Fig. 4:**
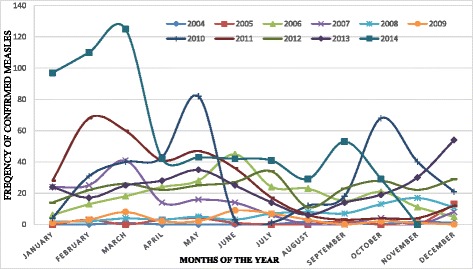
Trend of Measles Confirmed cases (%) by Age Group of Patients and Years in Amhara Regional State of Ethiopia, 2004–2014

Among 2412 lab confirmed measles, 1251 (51.9 %) were males and 9 cases had no information on sex available on the case report. The number of lab confirmed measles cases increased from 7 cases in 2004 to 611 in 2014 (Fig. 2). 1160 (48 %) of all lab-confirmed measles cases were in individuals ≥ 15 years of age (Fig. 4).

**Fig. 5:**
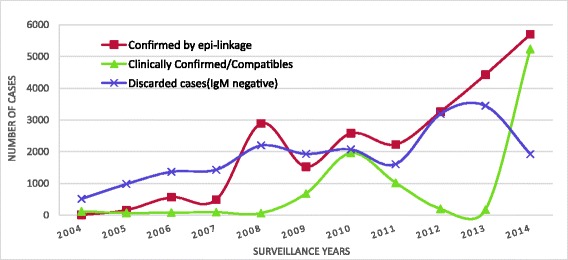
Measles Case Classification in Amhara Regional State of Ethiopia, 2004–2014

During the study period, the total number of clinically compatible cases was 9699. The percent and number of clinically confirmed/compatible cases fluctuated by year but reached its peak (35 %, 5237) in 2014. The proportion of discarded cases (measles IgM negatives) decreased dramatically from 70 % in 2004 to 12 % in 2014. 23,842 epidemiologically-linked (Epi-linked) cases were reported between 2004 and 2014. The number of Epi-linked cases reached its peak in 2014 at 5701 (Fig. 5).

## Discussion

The African region has set a goal to eliminate measles infection by 2020. This would require achieving a measles incidence of less than one confirmed measles case reported per million population per year. High measles vaccination coverage (95 % nationally and at district level), and a non-measles febrile rash (measles IgM negative or discarded case) rate of at least 2 per 100,000 population at national level indicating a high surveillance performance are two of the strategies needed to achieve this goal [2]. Although there is variability among zones, the Amhara Region State met the targets for these two principal measles surveillance performance indicators.

From January 2004 through December 2014, a total of 2412 laboratory confirmed measles cases were found from all the 11 zones of Amhara Regional State, Ethiopia. A total of 717 samples were not tested during 2009–2011 and in 2014 due to shortage of measles kit. During this same time period, 23,842 cases were classified as epidemiologically linked and an additional 9699 cases were defined as clinically compatible.

In general, the laboratory confirmed, clinically confirmed and epi-linked measles cases increased in the study period. The number and percentage of laboratory confirmed measles cases increased from 7 (5.7 %) in 2004 to 611 (65.9 %) in 2014 despite an increasing report of administrative measles vaccination coverage [11]. In the consistent laboratory testing system, the dramatic increase is likely due to a variety of factors including an increased pool of susceptibles due to long-standing sub-optimal vaccination coverage. The circulation of measles virus was affirmed by the presence of many confirmed outbreaks in the region [7, 8, 12]. The other contributing factor might be the improved surveillance system in terms of coverage, quality, and sensitivity due to massive health service expansion through time in the country as well in the region [13]. This finding was similar to the findings in Nigeria and Germany [3, 14].

We also noted a fluctuation of laboratory confirmed measles cases in the study years, high incidence of cases following a low incidence period detected in the area. This was a similar finding to a study in South West Nigeria [3], typical of measles epidemiology. Additionally, the interval and coverage of SIAs [15] conducted might have contributed to the fluctuation.

It was revealed from the result that there were a lower number of lab confirmed cases in 2009 relative to 2008 and 2010. This might be related to the high measles vaccination coverage (96 %) achieve during the SIA in the regional state during 2008 [16]. This finding was similar to the findings in Nigeria and Germany [3, 14] and reinforce the theory that measles is one of the first diseases to re-emerge when vaccination coverage falls [17].

With high coverage of routine measles vaccination, outbreaks are expected to stop altogether and transmission interrupted. However, in this region outbreaks did not stop and transmission was not interrupted despite increasing vaccination coverage reported in routine immunization and SIAs [15]. There were consistently increasing measles outbreaks like in South Gonder zone Simada district [12] and massive case reporting continued with increasing number of laboratory confirmed cases (Figs. 2, 4 and 5).

In this study, there was a gradual increase in the number of laboratory confirmed measles cases and persistent high measles positivity rate among patients ≥10 years age group and a decrease of cases among children under one year. This may reflect low immunization coverage in the region in previous years, and as a result more susceptibles accumulated in these age groups, which created a favorable condition for measles outbreaks to occur and measles to circulate in these age groups. On the other hand the lower positivity rate among <1 year children may indicate the improvement of routine immunization. This higher measles positivity rate among adults was similar finding with studies in South Africa [18] and Europe [19].

Measles infection in adults may have serious consequences for children. Infected adults will be unable to work and care for their children and will transmit the virus to their susceptible children [20] as measles is highly communicable, with secondary attack rates greater than 90 % among susceptible persons [1]. Although measles complications and deaths related to outbreaks were not captured in our database, there likely were deaths and complications that occurred in Amhara Regional State of Ethiopia during the study period as illustrated by other studies [11, 12].

In addition, our study revealed seasonality of measles confirmed cases during the period of analysis (2004–2014), with higher number of cases during February–March (hot dry season) and lower numbers in the rainy season of the region (July-September). This might be related with the population movement and many traditional ceremonies (wedding, religious festivals) during this season that created a favorable condition for measles transmission. Seasonal variation in the hot dry season was also noted in a study finding in Nigeria [3].

The analysis also indicated that a high proportion (60 %) of suspected measles cases reported and tested in this surveillance system had negative results for measles IgM throughout the study years. This high IgM negativity rate may be a good sign for the laboratory surveillance system.

Measles cases (suspected, confirmed) and outbreaks are increasing from year to year in the region. However, still there is no genotype data available to map circulating strain of the measles virus. Virus isolation and genotyping of measles virus to identify source of infection is one of the strategies recommended by WHO AFRO to achieve the measles elimination goal by 2020 [2].

The findings of our study are subjected to some limitations. First, not all measles suspected cases, especially during confirmed outbreaks, were reported through the laboratory case-based surveillance data. Only three laboratory confirmed measles were enough to declare a measles outbreak in that district; after that no additional samples are collected for the next one month in that district. Secondly, our results may not be representative of all cases of measles and may be biased toward zones that have a good surveillance system and populations with more access to care. Finally, our analysis is limited by the limited demographic and clinical information collected on each reported case.

## Conclusion and recommendation

In conclusion, the number and percentage of laboratory confirmed measles cases are increasing from year to year in the study area. Measles is widely circulating in the region primarily affecting age groups ≥10 years. We also noted a seasonal peak during February-May. Based on this finding, measles will remain a public health problem in the Amhara Regional State of Ethiopia unless concerted efforts are made in the region and implemented to increase the vaccination coverage and continuing sensitive case based surveillance performance.

We also recommend conducting a wide age group vaccination campaign in the region as there is more case in the age group >10 years. Additional study is needed in the region to better understand the age shift, and the knowledge, attitude and practices of the general population and health care professionals about measles infection and vaccination. As Ethiopia gets closer to measles elimination targets, it will be important to introduce genotyping to determine circulating measles virus strains.
